# Improved alignment quality by combining evolutionary information, predicted secondary structure and self-organizing maps

**DOI:** 10.1186/1471-2105-7-357

**Published:** 2006-07-25

**Authors:** Tomas Ohlson, Varun Aggarwal, Arne Elofsson, Robert M MacCallum

**Affiliations:** 1 Stockholm Bioinformatics Center, Stockholm University, SE-106 91 Stockholm, Sweden; 2 Department of Electrical Engineering and Computer Science, Massachusetts Institute of Technology, Cambridge, MA 02139, USA; 3 Center for Biomembrane Research, Stockholm University, SE-106 91 Stockholm, Sweden; 4 Division of Cell and Molecular Biology, Imperial College London, London, UK

## Abstract

**Background:**

Protein sequence alignment is one of the basic tools in bioinformatics. Correct alignments are required for a range of tasks including the derivation of phylogenetic trees and protein structure prediction. Numerous studies have shown that the incorporation of predicted secondary structure information into alignment algorithms improves their performance. Secondary structure predictors have to be trained on a set of somewhat arbitrarily defined states (e.g. helix, strand, coil), and it has been shown that the choice of these states has some effect on alignment quality. However, it is not unlikely that prediction of other structural features also could provide an improvement. In this study we use an unsupervised clustering method, the self-organizing map, to assign sequence profile windows to "structural states" and assess their use in sequence alignment.

**Results:**

The addition of self-organizing map locations as inputs to a profile-profile scoring function improves the alignment quality of distantly related proteins slightly. The improvement is slightly smaller than that gained from the inclusion of predicted secondary structure. However, the information seems to be complementary as the two prediction schemes can be combined to improve the alignment quality by a further small but significant amount.

**Conclusion:**

It has been observed in many studies that predicted secondary structure significantly improves the alignments. Here we have shown that the addition of self-organizing map locations can further improve the alignments as the self-organizing map locations seem to contain some information that is not captured by the predicted secondary structure.

## Background

The ability to create good alignments is important when inferring knowledge from one sequence to another. Alignments can be used in phylogeny to examine the evolution of sequences, and in protein structure prediction. In protein structure prediction, alignments are used to detect related sequences in a procedure called fold recognition, and to align the query sequence to related sequences.

In order to obtain good alignments, evolutionary information (multiple sequence alignments) should be used. It has also been shown that methods that use evolutionary information for both the query and target sequences are superior to methods that only use evolutionary information for either the query or the target sequence [1,2]. Methods that use evolutionary information for both the query and target sequences are known as profile-profile methods. Profile-profile methods have been observed to result in improved alignment quality compared to profile-sequence methods [3-8]. Even though profile-profile methods improve the alignments it has been observed that they do not necessarily provide optimal alignments [7].

To improve further both alignments and the detection of distantly related proteins, structural features of proteins are routinely used in the alignment procedure. Structural features that have been used include secondary structure [9,4,12] and solvent accessibility [13,14]. The secondary structure information has been incorporated into the score in different ways in different methods. In ORFeus [9] and prof_ss [11] a score based on the predicted secondary structure is added to the profile-profile score of FFAS [15] and prof_sim [16], respectively. Wang & Dunbrack constructed a secondary structure substitution table from predicted and observed secondary structures. The total score was calculated by adding the weighted *SS*_*score *to the weighted profile-profile score, where the sum of the two weights was set to one. However, this relationship between the profile-profile and the secondary structure score might not be optimal since only a modest improvement was seen in alignment quality. In contrast the addition of predicted secondary structure has been shown to improve the sensitivity of the *detection *of distantly related proteins significantly [9,11,4]. Finally Tang *et al*.'s hybrid sequence profile [12], using secondary structure and structural information, seems to be a good example where in particular secondary structure information improves both the homology detection as well as the alignment quality.

Although not completely uniform, a trend in studies to date suggest that predicted secondary structure improves alignments. It has also been shown that combining secondary structure predictions with other structural features can further improve the alignments [12,17]. This indicates that, if implemented correctly, different types of structural features can be included to improve alignment quality. We have recently developed a profile-profile scoring function, ProfNet [18]. In ProfNet the scoring function used in the subsequent alignment algorithm is trained to identify structurally superimposable residue pairs. ProfNet is based on an artificial neural network, which makes it easy to include additional information. Therefore, we wanted to investigate how an alternative way of representing structural information would affect the alignment quality. The structural information used in this study is captured by a self-organizing map (SOM). We found that the alignment quality can be improved for distantly related proteins by combining a profile-profile score with a SOM based score. The effect is not as great as when predicted secondary structure is included, but by combining these with the SOM score we were able to improve the alignments further.

## Results and discussion

### Incorporating local structural information into alignment scores

It is well known that secondary structure information has the power of improving fold recognition and alignments, but it is still not known how best to include it into an alignment score. In this study we used predicted secondary structure from PSI-PRED [19] for both the query and target sequences. First we constructed a normal profile-profile method using predicted secondary structures, which we refer to as Prob_score_SS. Here we use the same secondary structure scoring system as in ORFeus. This secondary structure score, *SS*_*score*, is weighted and added to Prob_score, which is our implementation of PICASSO3 [3], one of the best methods in our benchmark study of profile-profile methods [7]. Full details are provided in the Methods section.

Secondly, a new ProfNet version, ProfNet_SS, was constructed using the Prob_score score, and predicted secondary structure as input. Here, the *SS*_*score *was not calculated (as in Prob_score_SS), instead the six PSI-PRED propensity values (three from the query and three from the template) were used directly as input to the artificial neural network. Note that while the original ProfNet method takes two 20-dimensional profile vectors as input, we use the single Prob_score value as input for the new versions developed here.

**Figure 1:**
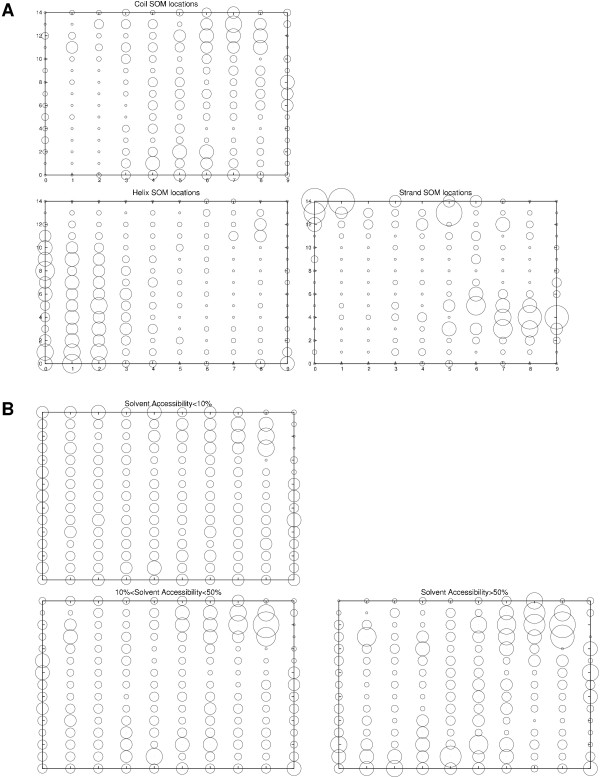
The mapping of (a) secondary structure states (helix, strand and coil) and (b) solvent accessibility to a 2D SOM trained using 15-residue windows of sequence profiles. The size of the circles is proportional to the number of times a sequence window having a particular secondary structure state is mapped to that position. The most populated helix and strand locations are almost non overlapping, while coil is found in most parts of the SOM and are somewhat avoiding the most popular helix locations. The total number of points in the three plots is 360 000 (130 000 in helix, 70 000 in strand and 160 000 in coil).

An alternative method to utilize local structural features is to cluster local similarities. Such a clustering can be done using a self-organizing map which maps high dimensional data into a 2D or 3D grid. In this study, the similarities in profile vectors of a large set of proteins are clustered in such a way as corresponding to local structure states (although note that only sequence information is used) [20]. An advantage of this approach is that it is not necessary to use predefined secondary structure states, instead the SOM clustering defines the states. In Figure 1a such a SOM mapping of a 15-residue sequence profile windows is shown. Interestingly, multiple distinct regions of helix, strand and, to some extent, coil can clearly be seen. In Figure 1b it can be seen that the SOMs also are able to capture some information about the solvent accessibility of the sequence windows, although this is less clear-cut (note the more overlapping regions of density). Although they capture aspects of secondary structure, it should be noted that SOMs are not able to predict three-state secondary structure as well as ANN based prediction methods, e.g. PSI-PRED (data not shown). In our subsequent studies, windows of profile vectors were mapped onto 3D SOM grids.

Preliminary studies suggested that the simultaneous use of SOM mappings based on different sequence window sizes produced slightly better results (data not shown). In this study we have used three SOMs trained with sequence profile windows of 7, 17 and 21 residues. Any given position in a sequence therefore maps to a 3D location in each of the three SOMs, making a total of 9 coordinates ("SOM locations").

**Figure 2:**
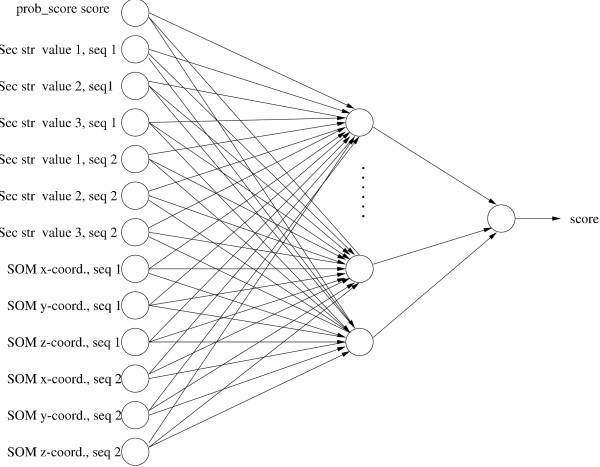
The architecture of the ANN used in ProfNet_SS_SOM. This example shows an ANN with Prob_score score, predicted secondary structure values and SOM locations as input. Only one SOM is shown for clarity, but note that three sets of SOM locations are used in ProfNet_SS_SOM (each one uses a different sequence window size).

Using this clustering technique we constructed two ProfNet versions, ProfNet_SOM, which uses Prob_score's score and SOM locations, and ProfNet_SS_SOM which uses Prob_score's score, predicted secondary structure and SOM locations as input, as depicted in Figure 2. For a summary of the inputs used in the different ProfNet methods, see Table 1.

For completeness, Prob_score_SOM and Prob_score_SS_SOM methods have also been implemented (see Methods for full details).

### Secondary structure information improves the alignments

The alignment quality performance was compared for protein pairs related at SCOP superfamily and fold level. The alignment quality of the Prob_score_SS method, as measured by the average MaxSub score, increased by 10% on superfamily level and by 40% on fold level (p-value 1·10^-5^) compared to Prob_score, see Table 2. This is in line with earlier results on the combination of predicted secondary structure with profile-profile scoring. It should also be remembered that Prob_score performs better than methods not using profile-profile scoring [18], i.e. the baseline for improvement is quite high.

ProfNet_SS was shown to produce alignments of similar quality as Prob_score_SS, with an improvement of 10 and 26% on superfamily and fold level (p-value 6·10^-4^) compared to ProfNet. Hence, a significant improvement in alignment quality could be seen on fold level by adding predicted secondary structure to Prob_score as well as to ProfNet, although the use of neural networks in this case is not particularly advantageous. These results show that predicted secondary structure is useful when aligning distantly related proteins, as observed in earlier studies [9-12].

### Combined secondary structure information and SOM locations further improve the alignments

**Figure 3:**
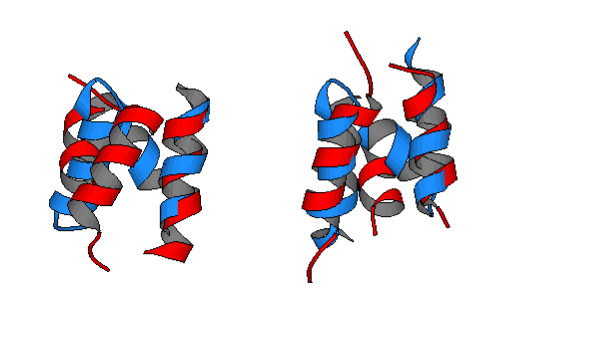
A superposition of the model structure (red) onto the experimental structure (SCOP code) dldv0a_.a.5.2.1 (blue). The model structure to the left is based on the alignment of ProfNet_SS and the model to the right on ProfNet_SS_SOM. The model is made using (SCOP code) d1eija_.a.5.6.1 as a template.

By combining plain SOM locations with Prob_score score in ProfNet (ProfNet_SOM) the alignments show a slight improvement by 5 and 8% on superfamily and fold level respectively compared to ProfNet. This improvement is not very impressive, however by combining predicted secondary structure information and SOM locations the improvement on fold level is 49% compared to ProfNet and 18% compared to ProfNet_SS (p-value 3·10^-2^). (A similar pattern is seen with Prob_score, Prob_score_SOM and Prob_score_SS_SOM.) This indicates that the SOM based classification and the PSI-PRED secondary structure predictions contain complementary information. Although the improvement of 18% might seem large it should be noted that this improvement is only seen at the fold level and the real increase in MaxSub score is small (0.02). Although we have optimized the parameters individually for each of the methods it was noted that the results were not extremely sensitive to this optimisation. Using 40 of the best parameter sets ProfNet_SS obtained an average MaxSub score of 0.073 ± 0.02 and ProfNet_SS_SOM 0.085 ± 0.02 (± standard deviation). This indicates that although the optimal parameter tuning improves the alignment quality by 10–20% the improvement from using the SOMs is consistent over a large set of parameters. Figure 3 shows an example where the addition of SOM information improves the protein model by aligning the helices more correctly.

### Why does the SOM information improve the alignments?

**Figure 4:**
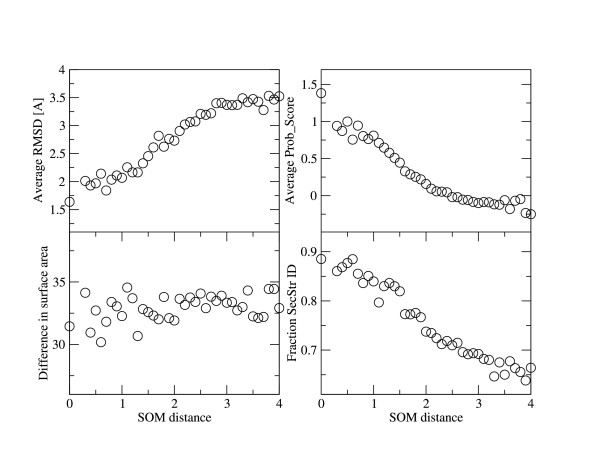
Average RMSD [Å], Prob_score, difference in solvent accessible surface area [Å^2^] and secondary structure identity is plotted against SOM distance (binned from 0–4 in steps of 0.1). The SOM distance is defined as the average of the distances between locations in the three SOMs used in ProfNet_SS_SOM, for structurally aligned residue pairs (i.e. those used as positive examples in the neural network training). It can be seen that among the pairs that are close in the SOM space the average distance in the structural superposition is closer and that a higher fraction of these pairs have the same secondary structure. The Prob_score is also (inversely) correlated with SOM distance. In contrast no such clear trend can be found for the difference in surface accessibility.

From the results in Table 2 we see that the alignments are improved most when a combination of profile-profile score, predicted secondary structure and SOM locations is used (ProfNet_SS_SOM), while ProfNet_SOM actually performs slightly worse than ProfNet_SS. To gain a deeper understanding into what the SOM locations capture we have analysed pairs of structurally aligned residues in terms of the "SOM distance" (explained below), structural distance (RMSD), Prob_score and secondary structure identity. The SOM distance is calculated as the Euclidean distance between the two points in the SOM cube to which a residue pair maps. More precisely it is the mean intra-SOM distance over the three SOMs which use different sequence profile window sizes. In Figure 4 it can be seen that residues pairs having similar SOM values (small SOM distance) are, on average, closer to each other in the structural superposition, that a higher fraction of these share the same secondary structure and that their sequence profiles are more similar. However, the average difference in solvent accessibility does not show a strong correlation to this measure. This indicates that the SOMs capture some information that might correspond to sequence/structure information that is not captured by secondary structure prediction or the profile-profile score. It might be speculated that the SOM clusters correspond to more fine grained features than the three-state model (helix, strand, coil) used in secondary structure predictions or to the existence of larger fragment with significant sequence structure correlation as used in a recent study to improve alignment qualities [21]. Alternatively subtle sequence signals that are not seen when adding the profile-profile scores might be captured as well.

## Conclusion

Here, we show that two different methods to combine predicted three-state secondary structure and profile-profile scores improve alignments for distantly related proteins. The two approaches are; addition of *SS*_*score *to Prob_score and by using the predictions directly from PSI-PRED in ProfNet_SS. Interestingly these two different approaches improve the alignments by a similar amount.

It was also found that predicted secondary structure combined with self-organizing maps (SOM) of sequence profile windows can be used to improve alignments of distantly related proteins (and perhaps unrelated analogous folds) by a further small amount. The SOMs appear to be capturing information that is not directly related to solvent accessibility and is partially orthogonal to predicted secondary structure. The clusters on the SOM may correspond to fine-grained secondary and supersecondary structures which appear to be conserved at the fold level.

## Methods

### Self-organizing maps

A set of SOMs were trained using 1029 randomly chosen protein domains from a subset of SCOP 1.57, where no two domains have more than 75% sequence identity. SOMs with two, three and four dimensions and sizes (9,6), (15,10), (30,15), (45,30), (5,6,7), (6,6,6), (4,4,4,4) were used in preliminary trials. The (5,6,7)-sized SOM performed marginally better than the others in the alignment quality test and was therefore used in the rest of the study. Similarly it was found that 10 training epochs were sufficient (50 and 100 epochs were also tested). Sequence profile windows of sizes of one to 21 centered around the residue in question were used as input to the SOMs.

The self-organizing map (SOM) of Kohonen and Makisara (1989) was used in this study, using the algorithm outlined below (as in MacCallum, 2004) [20], assuming *win*·20-dimensional "input" vectors, where *win *is the window size of profile vectors used, i.e. 1–21.

• Initialisation: create a 3D grid of size (5,6,7) of *win*·20-dimensional vectors, *v*, with random starting values

• Training: for each of 10 epochs:

- for each data point *x*:

1. find the closest grid vector, *v*_*winner*_, to point *x *according to an Euclidean distance measure

2. update *v*_*winner *_towards *x *by a small amount *α*, *v*_*winner *_← *v*_*winner *_+ *α*(*x *- *v*_*winner*_)

3. update neighbours of *v*_*winner *_within a certain radius *r *in the same way, but by a smaller amount

- reduce radius *r *and training rate *α*

• Application: any data point *x *can be assigned to a "winning" grid vector, *v*_*winner*_.

After training a SOM, any *win*·20-dimensional vector can be mapped to a position (*a*, *b*, *c*) on the grid. The result of the clustering is that data points which are close in the input space are mapped to the same or neighbouring grid nodes wherever possible. The SOM locations were normalized to (*a*/5, *b*/6, *c*/7) so the values are in the range zero to one before used as input to ProfNet_SOM and ProfNet_SS_SOM. Multiple SOM locations, using different SOM mappings corresponding to different profile window sizes were used as input to the neural network (see below).

### Artificial neural network training

ProfNet is based on a novel scoring function obtained by training an artificial neural network to recognise related residues. For the positive training examples, protein pairs from the same superfamily, but different families, were structurally aligned using STRUCTAL [22] and all pairs of residues within 3 Å separation were used. The negative training examples were created from randomly selected residue pairs of proteins from different folds. For the positive and negative data sets no more than 15 aligned positions from the same protein pair were used. The ANNs were trained to score the training examples according to the S-score [23] (S-score = 11+rmsd2/5
 MathType@MTEF@5@5@+=feaafiart1ev1aaatCvAUfKttLearuWrP9MDH5MBPbIqV92AaeXatLxBI9gBaebbnrfifHhDYfgasaacH8akY=wiFfYdH8Gipec8Eeeu0xXdbba9frFj0=OqFfea0dXdd9vqai=hGuQ8kuc9pgc9s8qqaq=dirpe0xb9q8qiLsFr0=vr0=vr0dc8meaabaqaciaacaGaaeqabaqabeGadaaakeaadaWcaaqaaiabigdaXaqaaiabigdaXiabgUcaRiabdkhaYjabd2gaTjabdohaZjabdsgaKnaaCaaaleqabaGaeGOmaidaaOGaei4la8IaeGynaudaaaaa@3815@). The *rmsd *is calculated between the *C*_*α *_atoms of the aligned residues. The artificial neural networks (ANNs) were trained on 80% of the dataset and the remaining 20% used as test set, where proteins from the same superfamily are only present in either the training or the test set, not both. The neural network package Netlab in MatLab was used for the ANN training [24,25]. A linear activation function was chosen, and the training was carried out using the scaled gradient algorithm. The training of the ANNs was done using a grid search over the number of hidden nodes and number of training cycles. After the initial grid search, the search procedure was tuned to the area that produced the highest MCC value on the test set. At least 49 sets of parameters were tested for each ANN. The ANN-based scoring function was chosen by selecting the ANN with the highest MCC-value and the minimum number of training cycles and hidden nodes. In the next step the ANN was used in the alignment quality test. The ANN scoring functions were implemented into the Palign [26] package, and called ProfNet.

### Alignment quality

The dataset used in the alignment quality test was also constructed from the same subset of SCOP version 1.57, class a to e, where no two protein domains have more than 75 % sequence identity. From this dataset we included no more than 5 proteins from the same superfamily and no more than one NMR model per domain target. In total 672 superfamily and 602 fold related protein pairs were included. In the superfamily related dataset, no proteins from the same family were included, and among the fold related protein pairs no proteins from the same superfamily were included. Throughout this study, only local alignments were used. For each alignment we created a model of the query protein and compared the structure of this model with the correct structure. The same dataset has been used in two earlier studies [7,18] where it was shown that ProfNet and Prob_score performed better than other profile methods. We used MaxSub [27] which finds the largest subset of *C*_*α *_atoms of a model that superimpose well over the experimental model. The results obtained using another method, LGscore [23], to measure the alignment quality were similar to using MaxSub and are not reported here.

### Statistical calculations

The p-values given in the text are calculated between the alignment quality scores generated from two methods (resulting in 602 different MaxSub scores when comparing the fold-related pairs), and gives an estimate of the probability that the two methods' results differ only by chance. Since we do not know what kind of distribution the alignment quality scores have we used the non-parametric Wilcoxon (sign rank) test using the program R [28]. We used the 0.05 p-value level as a threshold for statistical significance.

### Profiles

We used the log-odds profiles obtained after ten iterations of PSI-BLAST [29] version 2.2.2, using an E-value cutoff of 10^-3 ^and all other parameters at default settings. The search was performed against nrdb90 from EBI [30]. The frequency profiles, used in Prob_score, were back-calculated from the log-odds profiles obtained from PSI-BLAST as in [7]. The profiles used in the SOM clustering were created using the .mtx files from the 'makemat' program which are also used by the PSI-PRED program (see below).

### Secondary structure predictions

PSI-PRED version 2.0 [19] was used to predict the secondary structure using the .mtx files created as described in the 'Profiles' section above.

### Prob_score_SS

The predicted secondary structure score *SS*_*score *was calculated as in ORFeus;

*SS*_*score*_*i*,*j *_= *Σ*_*l *= {*H*,*E*,*C*}_*sec*_*str*1_*i*,*l*_·*sec*_*str*2_*j*,*l *_where *sec*_*str*1_*i*,*l *_is the predicted secondary structure of sequence one, residue number *i *to be in state *l*. The *SS*_*score *was added to the profile-profile method Prob_score's score. The weight for the *SS*_*score *was determined by linear combination of the *SS*_*score *and Prob_score's score. The weights were normalized so the profile-profile score had a weight of one. The score is then defined as,

*Prob*_*score*_*SS *= *Prob*_*score *+ *W·SS*_*score*

This method will be referred to as Prob_score_SS in the rest of the study.

### Prob_score_SOM and Prob_score_SS_SOM

In order to implement the Prob_score_SOM and Prob_score_SS_SOM methods, a single value is needed to describe the "SOM score" between two sequence positions. This is calculated as 11+SOM distance
 MathType@MTEF@5@5@+=feaafiart1ev1aaatCvAUfKttLearuWrP9MDH5MBPbIqV92AaeXatLxBI9gBaebbnrfifHhDYfgasaacH8akY=wiFfYdH8Gipec8Eeeu0xXdbba9frFj0=OqFfea0dXdd9vqai=hGuQ8kuc9pgc9s8qqaq=dirpe0xb9q8qiLsFr0=vr0=vr0dc8meaabaqaciaacaGaaeqabaqabeGadaaakeaadaWcaaqaaiabigdaXaqaaiabigdaXiabgUcaRiabbofatjabb+eapjabb2eanjabbccaGiabbsgaKjabbMgaPjabbohaZjabbsha0jabbggaHjabb6gaUjabbogaJjabbwgaLbaaaaa@3E86@. The SOM distance is described in the main text. The combination of scores follows the simple weighted sum as described above.

### Gap parameters

To obtain good alignment quality, the gap-opening, gap-extension and *shift *parameters have to be optimized individually for each method. The gap-parameters indicate how likely is it that a gap will be introduced (and extended) in the alignment, modeled using the affine gap-penalty. For all methods, the gap-parameters and *shift *values were calibrated using a grid of gap-opening (GO) and *shift *values. The gap-extension was set to be either 5 or 10% of the gap-opening penalty. We searched a grid of GO = (0.1,0.2...,0.5) and *shift *= (-0.5,-0.4,...,0.1) for the ProfNet methods, and GO = (0.2,0.3...,3.5) and *shift *= (-0.5,-0.45,...,1.5) for Prob_score_SS. The parameters were tuned toward the direction that produced the best results. The parameters with the highest average MaxSub score on fold level was taken from the results where the average MaxSub score on superfamily level were in the 95-th percentile. A majority of the ProfNet versions had gap-opening, gap-extension and *shift *values close to 0.3, 0.03 and -0.3.

## Authors' contributions

TO wrote the code for the analysis, designed the test set, performed the experiments for the alignments and wrote the manuscript. VA performed the test for the secondary structure prediction using the self-organizing maps. AE and RMM participated in the design of the study and collaborated in writing the manuscript. RMM implemented the code for the self-organizing maps. All authors have read and approved the manuscript.

## Figures and Tables

**Table 1:** The input data used by the ANNs in the different ProfNet methods.

Method	plain profiles	Prob_score score	pred. sec. str.	SOM locations
ProfNet	Y	N	N	N
ProfNet_SS	N	Y	Y	N
ProfNet_SOM	N	Y	N	Y
ProfNet_SS_SOM	N	Y	Y	Y

In ProfNet the "plain profile", i.e. the amino acid frequencies are used as the only input. In ProfNet_SS the sequence similarity is instead measured using the Prob_score measure and in addition six values from PSI-PRED predicted secondary structure probabilities are added. ProfNet_SOM is identical but the PSI-PRED values are replaced with the output from the SOM classification and in ProfNet_SS_SOM both outputs are combined.

**Table 2:** The alignment quality is measured by the average MaxSub score for protein pairs from the same SCOP superfamily (and different family) and fold (and different superfamily).

Method	Average MaxSub	
	
	Superfamily	Fold
Prob_score	0.20	0.063
Prob_score_SS	0.22	0.088
Prob_score_SOM	0.20	0.070
Prob_score_SS_SOM	0.22	0.093
ProfNet	0.20	0.072
ProfNet_SS	0.22	0.091
ProfNet_SOM	0.21	0.078
ProfNet_SS_SOM	0.22	0.107
